# Virus-like Particles Derived from Bacteriophage Beihai32 as a Versatile Carrier for Displaying Large Peptide Antigens

**DOI:** 10.3390/nano16150932

**Published:** 2026-07-29

**Authors:** Anna A. Zykova, Elena A. Blokhina, Marina A. Shuklina, Olga O. Ozhereleva, Sergey A. Klotchenko, Eugenia S. Mardanova, Nikolai V. Ravin

**Affiliations:** 1 Institute of Bioengineering, Research Center of Biotechnology of the Russian Academy of Sciences, 119071 Moscow, Russia; 2 Smorodintsev Research Institute of Influenza, Russian Ministry of Health, 197376 St. Petersburg, Russia

**Keywords:** virus-like particle, VLP vaccine, ssRNA bacteriophage, M2e peptide, influenza A virus, vaccine platform

## Abstract

Virus-like particles (VLPs) based on the capsid protein (CP) of the ssRNA bacteriophage Beihai32 represent a promising nanoscale platform for the presentation of heterologous peptides. Previous studies have shown that the C-terminus of the CP tolerates long insertions without compromising VLP assembly. Here, we demonstrate that the N-terminus is similarly permissive to extended insertions. Hybrid CPs with one to four copies of the influenza A virus M2e peptide fused to the N-terminus were expressed in *Escherichia coli*. Fusion proteins containing four copies of M2e self-assembled into spherical VLPs, displaying the inserted peptides on the surface. Subcutaneous immunization of mice with chimeric VLPs induced high titers of M2e-specific antibodies. Unlike C-terminal fusions, the N-terminal insertion prevented the induction of anti-carrier antibody response indicting masking of the carrier protein in the chimeric VLP. To evaluate the capacity of the N-terminus for larger inserts, green fluorescent protein (GFP, 238 a.a.) was attached to the N-terminus of CP. The hybrid protein was expressed in *Escherichia coli* and formed VLPs. GFP was displayed on the particle surface and retained fluorescent activity. Overall, the phage Beihai32 CP is a versatile platform for the presentation of peptide antigens, supporting its potential application in VLP-based vaccine design.

## 1. Introduction

Virus-like particles (VLPs), formed by viral capsid proteins and lacking, are considered one of the most powerful platforms for developing recombinant vaccines. They mimic the parental viruses in size, geometry, and ability to induce humoral and cellular immune responses [1,2]. Moreover, VLPs are safe because they do not contain the pathogen’s genetic material. VLPs can also serve as platforms to display heterologous antigens, resulting in the formation of chimeric VLPs. The inclusion of antigens in the chimeric VLP increases their immunogenicity when they are presented on the surface of the particle [2].

VLPs based on capsid proteins of ssRNA bacteriophages represent one of the most promising platforms for antigen display and vaccine development. These bacteriophages, belonging to the family *Fiersviridae* (formerly known as *Leviviridae*), are characterized by compact genomes and relatively simple capsid architectures, making them convenient systems for genetic and chemical modification. The genomes of *Fiersviridae* phages consist of single-stranded RNA of approximately 3–4 kb in length, encapsulated within an icosahedral protein capsid with a diameter of ~28 nm. The capsid is composed of 178 copies of the capsid protein (CP) and a single copy of the maturation protein (Mat), which mediates the attachment of the viral particle to bacterial pili and facilitates the delivery of the genomic RNA into the host cell [3,4].

The expression of capsid proteins of these bacteriophages in bacterial systems typically results in the efficient self-assembly of VLPs without the need for additional components. Target antigens can be genetically fused to the CP, resulting in their surface display on assembled chimeric VLPs. Currently, VLPs based on capsid proteins of ssRNA bacteriophages such as Qβ, MS2, AP205, PP7, and P22 are used in vaccine development [2]. VLPs derived from the MS2 capsid protein were extensively employed for the display of antigens of diverse origin. In particular, they have been used for the presentation of foot-and-mouth disease virus antigens [5] as well as epitopes of human papillomaviruses [6,7], demonstrating the ability to induce specific immune responses. VLPs based on the PP7 capsid protein are also used for the presentation of human papillomavirus antigens [8]. In addition, AP205-based VLPs have been applied for the presentation of coronavirus antigens [9] and as carriers of conserved influenza A virus M2e peptides [10].

The tolerance of capsid proteins from different bacteriophages to insertions of heterologous sequences varies considerably. Incorporating a foreign peptide into the CP can alter its spatial structure and disrupt the fusion protein’s ability to self-assemble into VLP. The target peptide may also not be presented on the VLP surface, reducing its immunogenicity. Increasing the length of the foreign peptide typically leads to more pronounced structural disruptions in the fusion protein. For example, VLPs based on the phage AP205 CP can accommodate terminal modifications, with insertions of up to ~55 a.a. tolerated at the C-terminus and up to 39 a.a. at the N-terminus [11]. Similarly, the assembly of MS2-based VLPs is highly dependent on the size of the inserted fragment. It has been shown that the formation of morphologically and functionally intact particles occurred when the insertion length did not exceed 91 a.a. [12].

The limited number of bacteriophage capsid proteins exhibiting high tolerance to structural modifications significantly restricts the range of antigens that can be efficiently displayed on phage-derived VLPs. Therefore, the search for new bacteriophage CPs capable of self-assembly and accommodating peptide insertions of varying length and structural complexity is of particular importance.

The rapid advancement of high-throughput sequencing technologies has greatly expanded the discovery of previously uncharacterized bacteriophages, including those with ssRNA genomes, which can be considered potential sources of capsid proteins for the construction of VLPs. For example, metagenomic studies have identified more than 100 novel ssRNA bacteriophages, and it has been demonstrated that capsid proteins from 80 of them are capable of self-assembly into VLPs upon expression in *E. coli* [13].

We selected the CP of the ssRNA bacteriophage Beihai32, consisting of 130 amino acids (14.3 kDa), as a platform for VLP design. Structure superposition-based analysis revealed a similarity between the Beihai32 CP and AP205-like CPs [14]. The spatial organization of the N- and C-terminal regions of the CP is a critical factor in the design of chimeric VLPs, as it determines the possibility of using these regions to insert foreign peptides without disrupting VLP assembly. In the case of the phage Beihai32, both the N- and C-termini of CP are oriented outward, consistent with structures reported for other AP205-like phages [14,15]. This topology provides accessibility for the incorporation of heterologous peptide sequences, making Beihai32 a promising platform for recombinant VLP development.

Previously, we generated VLPs based on the Beihai32 capsid protein displaying four copies of the influenza A virus M2e peptide at the C-terminus. These chimeric CPs, produced in *Escherichia coli*, formed VLPs of approximately 30 nm in diameter, that induced high levels of M2e-specific antibodies upon immunization of mice and conferred protection against lethal influenza virus challenge [16]. Furthermore, it was demonstrated that the incorporation of a conserved region of the hemagglutinin HA2 subunit at the C-terminus of this recombinant protein did not impair VLP formation [17], with the insert length reaching 177 a.a. Moreover, it has also been demonstrated that the C-terminus of the Beihai32 CP can tolerate the incorporation of even longer heterologous sequences without compromising protein solubility or VLP self-assembly, as exemplified by 238 a.a long green fluorescent protein (GFP) insertion [18].

At the same time, the possibility of using the N-terminus of Beihai32 CP to attach foreign antigens remains poorly characterized. A single study reported that insertion of three copies of the influenza M2e peptide at the N-terminus prevented the expression of the recombinant protein *E. coli* [19].

In this study, we investigated the potential of the N-terminal region of the Beihai32 CP for incorporation of foreign proteins, using tandem repeats of the influenza A virus M2e peptide (insertion length 23–119 a.a.) and GFP as model inserts. We constructed several fusion proteins and found that the N-terminus of the CP allows for the incorporation of four copies of the M2e peptide and the GFP protein without disrupting the assembly of the chimeric VLPs, with the target proteins exposed on the particle surface. Furthermore, immunization of mice with chimeric VLPs carrying four copies of the M2e peptide induced high antibody titers against the target antigen and a weak immune response against the carrier protein, which may enable the reuse of this platform.

## 2. Materials and Methods

### 2.1. Bacterial Strains and Expression Vectors

For genetic manipulations and recombinant protein expression, the *E. coli* strain DLT1270, a derivative of DH10B carrying a chromosomally integrated *lacI* gene encoding the lac operon repressor was used. Recombinant protein expression was performed using the plasmid vectors pQE30 and pQE60 (Qiagen, Hilden, Germany).

### 2.2. Synthetic Oligonucleotides

Synthetic oligonucleotides F_EcoRV,ApaI-Beihai (TAT AGA TAT CGG GCC CTC AAA ACC AAT TGC TAT TTT), and R_Beihai-HindIII (TAT AAA GCT TTT CAG TGA TGA CAA GAT CCT) were used.

### 2.3. Construction of Expression Vectors

The pQE60 Beihai32 and pQE30 his-19S-GFP vectors for expression of empty Beihai32 CP and 19S-GFP proteins, respectively, were described previously [16,18].

#### 2.3.1. Vector for Expression of N-Terminal GFP-Beihai32 CP Fusion Protein

The DNA sequence encoding the Beihai32 CP gene was amplified by PCR using the primers F_EcoRV,ApaI-Beihai and R_Beihai-HindIII, with plasmid pQE60 Beihai32 [16] DNA as a template. The resulting PCR fragment was cloned into the ApaI and HindIII sites of pQE30 his-19S-GFP-19S-PQ465 [20], replacing the PQ465 CP coding sequence. The resulting vector pQE30 his-19S-GFP-19S-Beihai32 encodes GFP fused to the N-terminus of the Beihai32 CP via a flexible glycine-serine 19S linker (GTSGSSGSGSGGSGSGGGG) [21].

#### 2.3.2. Vectors for Expression of N-Terminal M2e-Beihai32 CP Fusion Proteins

The sequence of the M2e peptide matched the consensus sequence from human influenza A strains [22] with two cysteine substitutions for serines to prevent the formation of disulfide bonds and protein aggregation (SLLTEVETPIRNEWGSRSNDSSD, positions of Cys to Ser changes underlined).

DNA fragments encoding one, two, three, or four copies of the M2e peptide fused to the C-terminus of the 19S linker (19S-xM2e) were excised by digestion with BamHI and EcoRV from plasmids pQE60 HBc/19S-1M2e-19S, pQE60 HBc/19S-2M2e-19S, pQE60 HBc/19S-3M2e-19S, and pQE60 HBc/19S-4M2e-19S [23], and cloned into the corresponding sites of pQE30 his-19S-GFP-19S-Beihai32 plasmid. This resulted in the expression vectors pQE30 his-19S-1M2e-Beihai32, pQE30 his-19S-2M2e-Beihai32, pQE30 his-19S-3M2e-Beihai32, and pQE30 his-19S-4M2e-Beihai32, encoding one to four copies of the M2e peptide at the N-terminus of the Beihai32 CP.

DNA fragments encoding one to four copies of the M2e peptide flanked by 19S linkers from both sides (19S-xM2e-19S) were obtained by digestion with BamHI and ApaI from the same plasmids (see above) and cloned into the corresponding sites of pQE30 his-19S-GFP-19S-Beihai32. The resulting vectors pQE30 his-19S-1M2e- 19S-Beihai32, pQE30 his-19S-2M2e-19S-Beihai32, pQE30 his-19S-3M2e-19S-Beihai32, and pQE30 his-19S-4M2e-19S-Beihai32 encoded one to four copies of the M2e peptide, separated by 19S linkers from the N-terminal hexahistidine tag and from the N-terminus of the Beihai32 CP.

#### 2.3.3. Vectors for Expression of C-Terminal Beihai32 CP-M2e Fusion Proteins

To construct pQE60 Beihai32-19S-4M2e-his plasmid, the DNA sequence encoding the Beihai32 CP was excised by digestion with EcoRI and BamHI from vector pQE60 Beihai-19S-GFP-his [18] and cloned at the corresponding sites of pQE60 PQ465-19S-4M2e-his [16], replacing the sequence encoding the PQ465 CP. The resulting vector encoded the Beihai32 CP, followed by a 19S linker and four copies of the M2e peptide.

### 2.4. Expression of Recombinant Proteins in E. coli

The expression vectors were introduced into *E. coli* DLT1270 cells. Recombinant *E. coli* strains were grown at 37 °C in LB medium supplemented with 100 μg/mL ampicillin until the mid-log phase (*OD*600 ~ 0.5). Protein expression was induced by the addition of isopropyl β-D-1-thiogalactopyranoside (IPTG) to a final concentration of 1 mM, followed by incubation at 20 °C overnight with shaking. After induction, cells were harvested by centrifugation at 4000 *g* for 15 min. The cell pellet was resuspended in 10 mM phosphate-buffered saline (PBS, 10 mM Na-phosphate buffer, 150 mM NaCl pH 7.2) containing lysozyme (1 mg/mL) and incubated at room temperature for 15 min. The cell suspension was frozen at −20 °C overnight, thawed, and lysed by sonication on ice (Bandelin SONOPULS, Bandelin Electronic GmbH, Berlin, Germany; HD 2200 mode, 10% cycle). The suspension was centrifuged at 12,000 *g* for 10 min. The supernatant was collected, and the pellet was resuspended in an equivalent volume of PBS.

Expression of Beihai32 and 19S-GFP proteins was performed as described previously [16,18].

Protein electrophoresis was performed in polyacrylamide gel (PAGE, 10–12%) in the presence of sodium dodecyl sulfate (SDS).

### 2.5. Purification of Recombinant Proteins

VLPs based on 4M2e-Beihai32 and 4M2e-19S-Beihai32 proteins were purified under native conditions from the soluble fraction of the cell lysate using metal-affinity chromatography on Ni-NTA-agarose (Qiagen, Hilden, Germany). His-tagged proteins were bound to Ni-NTA agarose in 10 mM PBS pH 7.2 with 1M NaCl. Unbound proteins were removed by washing with the same buffer supplemented with 16 mM imidazole. The target proteins were eluted with PBS containing 500 mM imidazole.

For Beihai32-19S-4M2e and GFP-19S-Beihai32 VLPs, an initial purification step involved precipitation with 25% saturated ammonium sulfate at 4 °C. The precipitate was collected by centrifugation at 13,000 *g* and resuspended in 10 mM PBS containing 1 M NaCl. Furthermore, the VLPs were purified using metal-affinity chromatography on Ni-NTA-agarose (Qiagen, Hilden, Germany) under native conditions. Protein binding was carried out for 1 h in 10 mM PBS containing 1 M NaCl. Washing and elution steps were performed as described above.

VLPs formed by Beihai32 CP were purified by ultracentrifugation (35,000 rpm at 20 °C, 22 h, SW40 rotor, Optima L-90K (Beckman Coulter, Indianapolis, IN, USA)) of the cell lysate in a sucrose–cesium chloride density gradient [16]. Recombinant GFP was purified under native conditions from the soluble fraction of the cell lysate using metal affinity chromatography on Ni-NTA-agarose (Qiagen, Hilden, Germany) as described previously [18]. After purification, all proteins were dialyzed against 10 mM PBS.

Experiments on expression of recombinant proteins and purification of VLPs were performed at least 3 times; the results were reproducible.

### 2.6. Analysis of the VLP Structure

The structure of VLPs was analyzed by transmission electron microscopy (TEM) and dynamic light scattering (DLS). TEM was performed using a JEM 1400 transmission electron microscope (JEOL, Tokyo, Japan). Protein samples were placed on formvar/carbon-coated copper grids (TED PELLA, Redding, CA, USA) and stained with a 1% (*w*/*v*) uranyl acetate in methanol. The hydrodynamic diameter of the particles was determined by DLS using a Zetasizer Nano S90 particle size analyzer (Malvern Panalytical, Great Malvern, UK). Measurements were carried out at 25 °C in square polystyrene cuvettes (12 mm; DTS0012).

### 2.7. Analysis of Antigenic Properties of VLPs by ELISA

ELISA plates were coated with serial dilutions of VLPs (Beihai32, 4M2e-Beihai32, 4M2e-19S-Beihai32, and Beihai32-19S-4M2e) in sodium bicarbonate buffer (pH 8.5) overnight at 4 °C. After washing with PBST (PBS containing 0.05% Tween-20), the plates were treated with a blocking buffer (0.2% (*w*/*v*) BSA in PBS) for 1 h at 37 °C. Following one wash with PBS, plates were incubated with mouse polyclonal antibodies against M2e or Beihai32 CP for 30 min at 37 °C. Plates were then washed five times with PBST. Horseradish peroxidase (HRP)-conjugated anti-mouse secondary antibodies (IMTEK, Moscow, Russia) diluted 1:10,000 were added and incubated for 30 min. After five washes with PBST, tetramethylbenzidine (TMB; Vector-Best, Novosibirsk, Russia) was used as the substrate. The reaction was stopped by the addition of 0.5 N HCl, and absorbance was measured at 450 nm using a Multiskan FC microplate reader (Thermo Fisher Scientific, Waltham, MA, USA).

In the case of GFP-based proteins, ELISA plates were coated with serial dilutions of GFP, GFP-19S-Beihai32, Beihai32 in carbonate–bicarbonate buffer (pH 8.5) overnight at 4 °C. Protein concentrations were adjusted based on the equimolar content of GFP or Beihai32 present in the protein molecule. Twofold serial dilutions of GFP-19S-Beihai32 (starting from 15 µg/mL) and GFP (starting from 10 µg/mL) were applied to the plates, followed by washing and blocking steps as described above, and incubation with rabbit monoclonal anti-GFP antibodies. In another experiment twofold dilutions of GFP-19S-Beihai32 (starting with 30 μg/mL) and Beihai32 (starting with 10 μg/mL) proteins were applied to the plates, which were then blocked, washed and incubated with mouse polyclonal antibodies against Beihai32 CP. HRP-conjugated anti-rabbit (Promega, Madison, WI, USA) or anti-mouse secondary antibodies (IMTEK, Moscow, Russia) were used for detection. TMB (Vector-Best, Novosibirsk, Russia) served as the substrate, and the reaction was stopped with 0.5 N HCl. Absorbance was measured at 450 nm using a Multiskan FC microplate reader (Thermo Fisher Scientific, Waltham, MA, USA).

### 2.8. Immunogenicity Analysis

Female BALB/c mice (haplotype H-2d) (16–18 g, 5 per group) were subcutaneously immunized three times at two-week intervals with VLPs formed by Beihai32, 4M2e-19S-Beihai32, and Beihai32-19S-4M2e proteins at a dose of 50 μg per mouse formulated with aluminum hydroxide adjuvant (Alum; #A8222, Sigma-Aldrich, St. Louis, MO, USA). Control groups received subcutaneous injections of empty Beihai32 CP particles or adjuvant solution or PBS.

On the 14th day after the third immunization, serum samples were collected from five mice in each group to assess anti-M2e and anti-CP antibody titers. Antibody titers were measured using enzyme-linked immunosorbent assay (ELISA). 96-well microtiter plates (Greiner, Pleidelsheim, Germany) were coated with the synthetic peptide G37 corresponding to the consensus M2e sequence of human influenza A strains (SLLTEVETPIRNEWGCRCNDSSD) (5 μg/mL) or purified Beihai32 CP (2 μg/mL). HRP-conjugated goat polyclonal anti-mouse IgG antibodies (Abcam, Cambridge, UK) were used at a dilution of 1:5000. Tetramethylbenzidine (Hema, Moscow, Russia) was used as a substrate. The reaction was stopped with 2N H_2_SO_4_, and absorbance was measured at 450 nm using a microplate reader Multiskan Skyhigh (Thermo Fisher Scientific, Waltham, MA, USA). The antibody titer was determined as the highest serum dilution with an OD_450_ at least twice that of the blank’s average value.

### 2.9. Statistical Analysis

Statistical analysis was performed using GraphPad Prism v.10.4.0. Differences between groups in ELISA-derived anti-M2e antibody titers were evaluated by one-way analysis of variance (ANOVA) followed by Tukey’s multiple comparisons test.

### 2.10. Ethics Statement

The study was carried out according to the recommendation of the Board of the Eurasian Economic Commission “On the Guidelines for working with laboratory (experimental) animals when conducting preclinical (non-clinical) studies” (14 November 2023, No 33). The experiments were approved by the Bioethics Committee on the Use of Animals of the Smorodintsev Research Institute of Influenza (Permit ID 24 dated 29 August 2024). All possible efforts were made to minimize the suffering of the animals.

## 3. Results

### 3.1. Design, Expression, and Purification of Recombinant Beihai32 CPs Carrying from One to Four Copies of M2e

**Figure 1:**
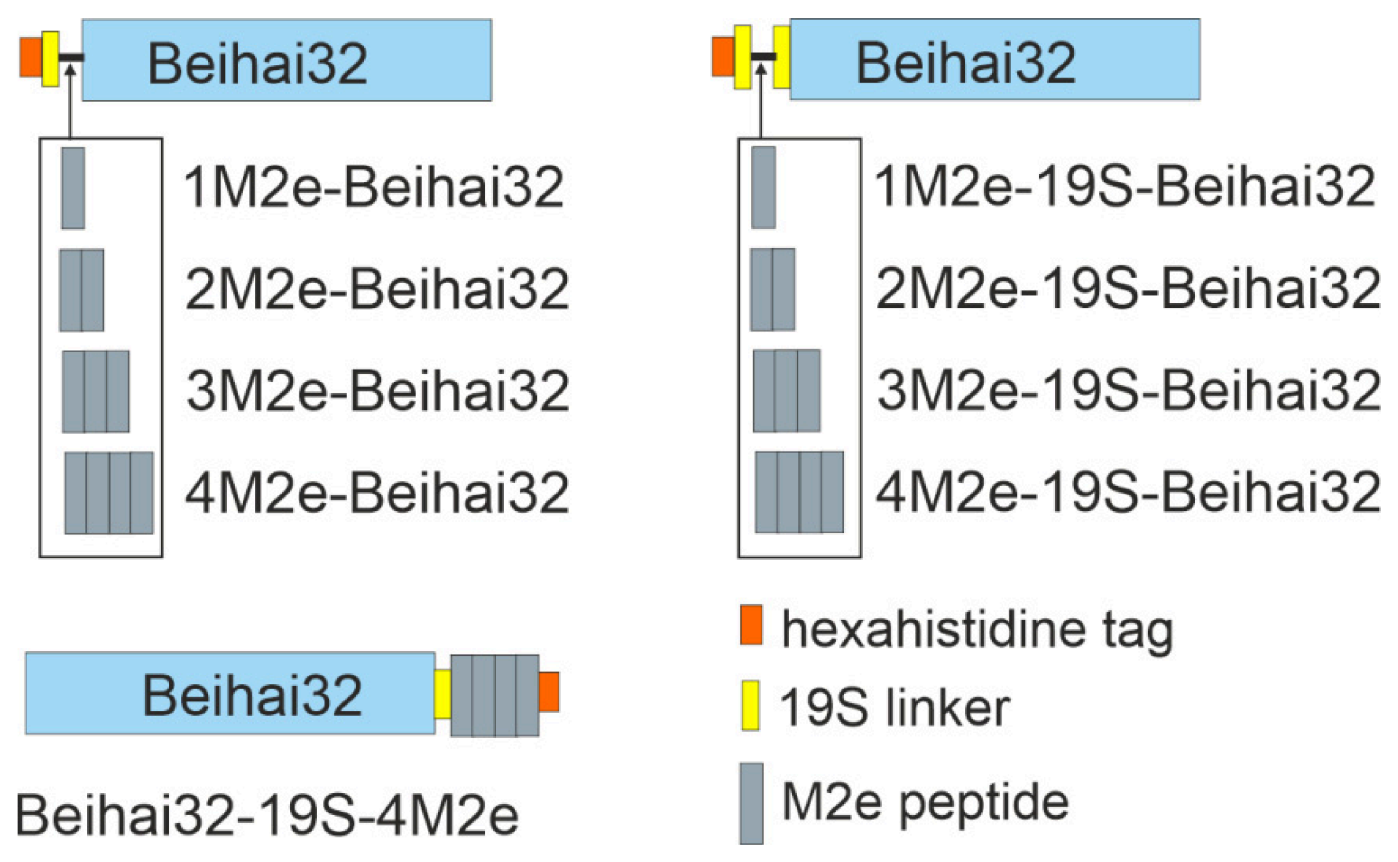
Structures of the recombinant proteins comprising M2e. Beihai32, CP of bacteriophage Beihai32.

To evaluate the ability of the Beihai32 CP to accommodate N-terminal insertions, the M2e peptide of influenza A virus was selected as a model antigen. The M2e peptide was incorporated into the fusion protein in the form of one, two, three, or four tandem repeats. In the M2e sequence, cysteine residues at positions 17 and 19 were substituted with serine to prevent disulfide bond formation and protein aggregation. This modification has been reported not to affect M2e immunogenicity [22]. One to four copies of the M2e peptide were genetically fused to the N-terminus of the Beihai32 CP either directly or via a flexible glycine-serine linker (19S). The resulting genes were cloned into the pQE30 vector, allowing the expression of recombinant proteins with an N-terminal His-tag (Figure 1).

For comparative analysis, four copies of the M2e peptide were also fused to the C-terminus of the Beihai32 CP via a 19S linker. The corresponding gene was cloned into the pQE60 vector to express the recombinant protein with a C-terminal His-tag (Figure 1).

**Figure 2:**
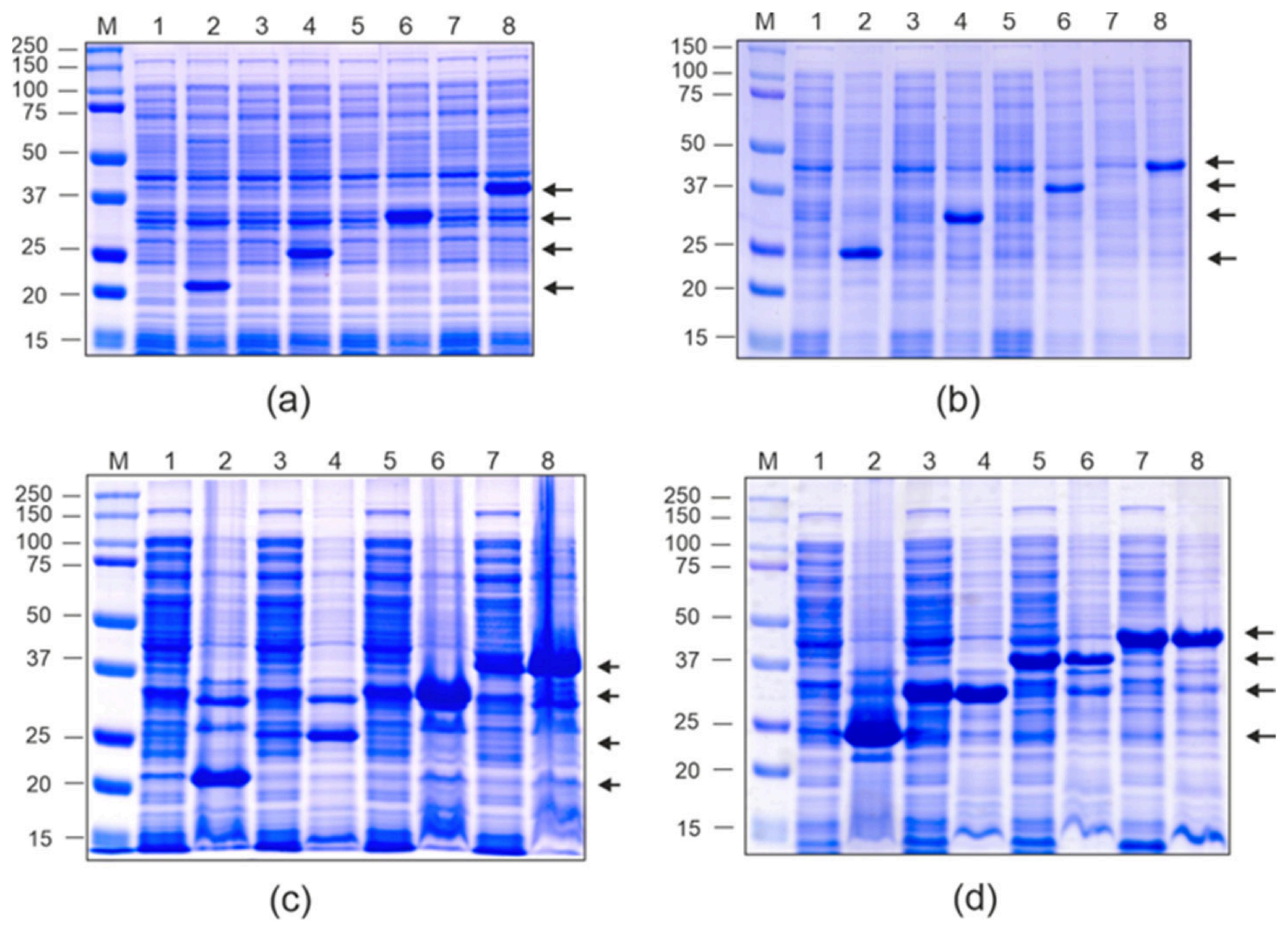
Expression of recombinant proteins in *E. coli*. Proteins isolated from *E. coli* cells were analyzed by SDS-PAGE. M, molecular weight marker (kDa). (**a**,**b**) total protein samples isolated from of *E. coli* cells before (lanes 1, 3, 5, 7) and after (lanes 2, 4, 6, 8) induction of expression. (**c**,**d**) fraction of soluble (lanes 1, 3, 5, 7) and insoluble proteins (lanes 2, 4, 6, 8). Proteins: (**a**,**c**): 1M2e-Beihai32 (lanes 1 and 2), 2M2e-Beihai32 (lanes 3 and 4), 3M2e-Beihai32 (lanes 5 and 6), 4M2e-Beihai32 (lanes 7 and 8); (**b**,**d**): 1M2e-19S-Beihai32 (lanes 1 and 2), 2M2e-19S-Beihai32 (lanes 3 and 4), 3M2e-19S-Beihai32 (lanes 5 and 6), 4M2e-19S-Beihai32 (lanes 7 and 8. The positions of target proteins are indicated by arrows.

All recombinant proteins were expressed at high levels in the *E. coli* (Figure 2). Following induction, protein samples were isolated from the soluble and insoluble fractions of the cell lysate and analyzed by SDS-PAGE. 1M2e-Beihai32 protein was detected almost exclusively in the insoluble fraction, whereas 2M2e-Beihai32, 3M2e-Beihai32, and 4M2e-Beihai32 were present in both soluble and insoluble fractions (Figure 2). Proteins comprising the 19S linker between the CP and M2e were mostly localized in the soluble fraction, with the exception of 1M2e-19S-Beihai32, which remained insoluble (Figure 2). Thus, the introduction of the 19S linker between the Beihai32 CP and the insert, as well as insertion of more than one copy of M2e, had a positive effect on the solubility of the recombinant fusion proteins.

Fusion proteins containing four copies of the M2e peptide were selected for further analysis because they represented the longest insert, and the inclusion of multiple copies of M2e in the fusion protein that forms VLPs enhances the immune response to M2e [23]. Recombinant proteins 4M2e-Beihai32 and 4M2e-19S-Beihai32 were purified from the soluble fractions of cell lysates (Supplementary Figure S1).

### 3.2. Recombinant Beihai32-Based Proteins with N- or C-Terminal M2e Insertions Form VLPs

**Figure 3:**
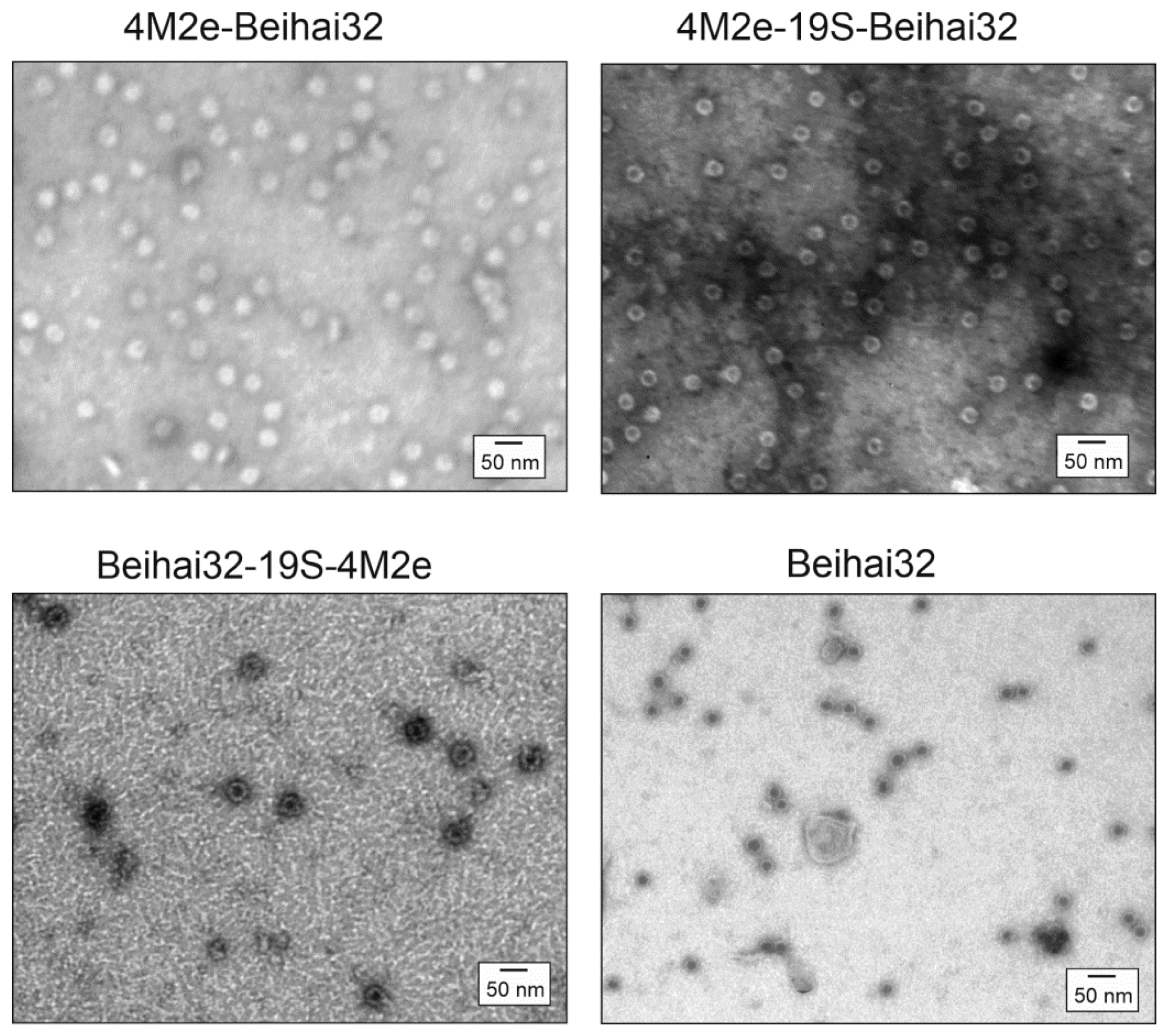
Analysis of VLPs formed by recombinant proteins using TEM.

The assembly of recombinant proteins 4M2e-Beihai32, 4M2e-19S-Beihai32, Beihai32-19S-4M2e and Beihai32 (empty CP) into VLPs was analyzed by dynamic light scattering (DLS) and transmission electron microscopy (TEM). According to DLS measurements, purified samples of 4M2e-Beihai32, 4M2e-19S-Beihai32, and Beihai32-19S-4M2e proteins contained particles with an average size of approximately 30–45 nm (Table 1). Likewise, TEM analysis revealed spherical VLPs with a diameter of approximately 30–35 nm (Figure 3). The Beihai32 CP without insertions formed particles with a diameter of approximately 28 nm. Thus, the presence of N- or C-terminal insertions of up to 119 a.a. in the Beihai32 CP does not interfere with VLP formation.

### 3.3. Surface Display of the M2e Peptide on Recombinant VLPs

**Figure 4:**
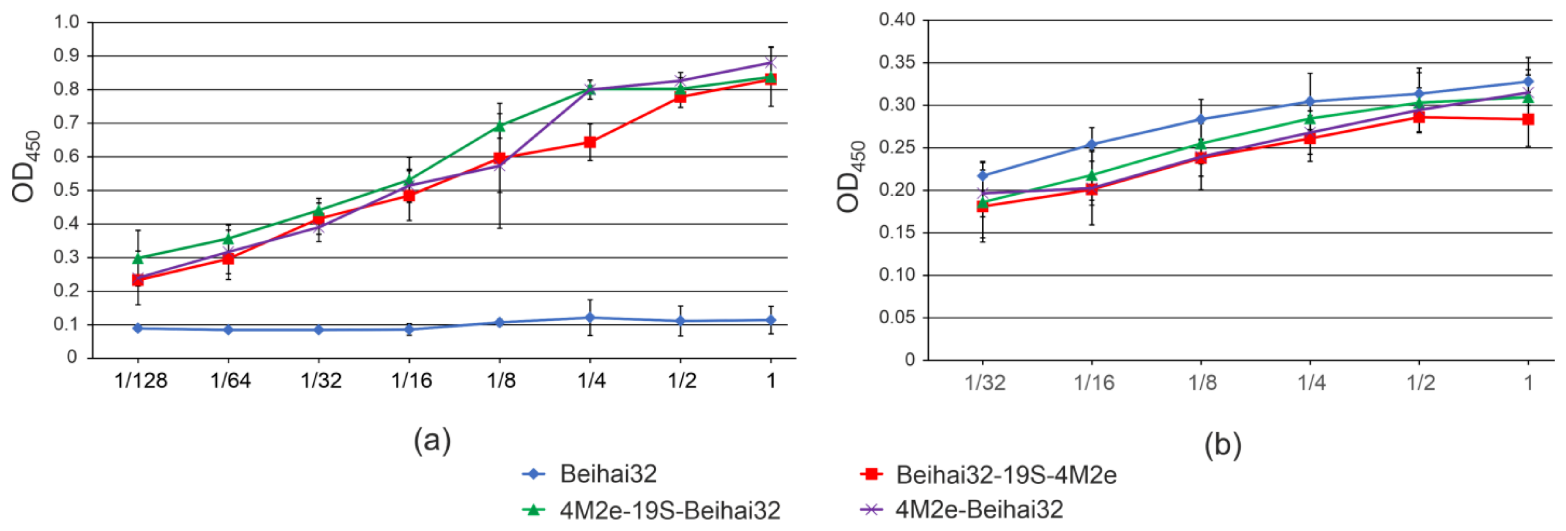
Antigenic characteristics of VLPs. Two-fold dilutions of VLPs formed by Beihai32, Beihai32-19S-4M2e, 4M2e-Beihai32, 4M2e-19S-Beihai32 proteins were loaded on ELISA plates and probed with antibodies against M2e (**a**) or Beihai32 CP (**b**).

A critical parameter determining the immunogenicity of chimeric VLPs is their spatial organization, specifically the presentation of antigens on the particle surface. The antigenic properties of recombinant VLPs containing four copies of the M2e peptide (4M2e-Beihai32, 4M2e-19S-Beihai32 and Beihai32-19S-4M2e) were evaluated by ELISA using anti-M2e antibodies (Figure 4). These antibodies bound with comparable efficiency to all chimeric VLPs, regardless of presence of the 19S linker and whether the insert was located at the N- or C-terminus of the capsid protein. These results indicate that the M2e peptide is exposed on the surface of the recombinant VLPs and retained accessible to specific antibodies.

At the same time, complete masking of the CP core of chimeric VLPs was not observed, as evidenced by the binding of chimeric VLPs to antibodies against “empty” Beihai32 capsid, although with lower efficiency compared to VLPs formed by the unmodified Beihai32 capsid (Figure 4).

### 3.4. Immunogenicity of Chimeric VLPs

Based on the conducted experiments, no clear advantage was observed for the insertion of the 19S linker between the CP and the 4M2e insert in the recombinant proteins 4M2e-Beihai32 and 4M2e-19S-Beihai32. Both proteins exhibited similar characteristics, suggesting that the inclusion of the linker does not significantly enhance protein expression, solubility, or VLP assembly. Therefore, for subsequent immunogenicity evaluation, 4M2e-19S-Beihai32 particles were selected for comparison with Beihai32-19S-4M2e particles containing a similar 4xM2e insert at the C-terminus.

**Figure 5:**
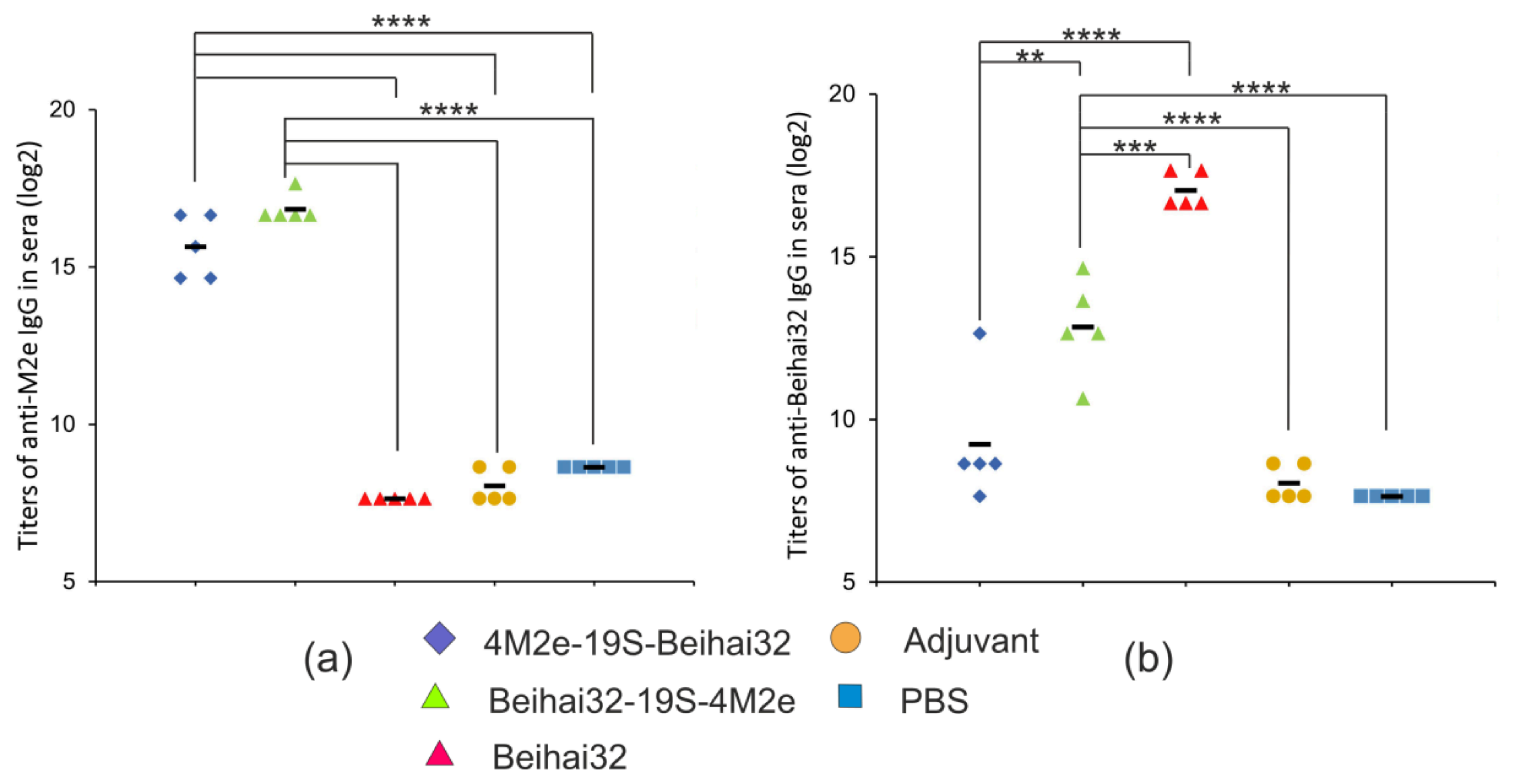
Immunogenicity of chimeric VLPs in mice: induction of anti-M2e antibodies. ELISA was used to determine the titers of anti-M2e IgG (**a**) and anti-Beihai32 CP IgG (**b**) in sera of mice (5 per group). Mean titers and endpoint titers (log2) observed in individual mice are shown. Statistically significant differences between groups are indicated (**: *p* < 0.01; ***: *p* < 0.001; ****: *p* < 0.0001).

Mice were immunized subcutaneously with analyzed VLPs in the presence of aluminum hydroxide adjuvant. Blood sera were taken after the third immunization and analyzed by ELISA to detect M2e-specific IgG antibodies. Immunization with Beihai32-based recombinant proteins induced high levels of M2e-specific antibodies in sera after the third immunization (Figure 5a). No statistically significant differences in anti-M2e antibody titers were observed between groups immunized with VLPs formed by N- or C-terminal 4M2e insertions.

Immunization with chimeric VLPs also resulted in the induction of antibodies specific to the carrier CP (Figure 5b). Immunization with the Beihai32 CP particles induced significantly higher levels of anti-CP antibodies compared to both types of chimeric VLPs containing the influenza A virus M2e peptides. Furthermore, a statistically significant difference was observed between mice immunized with 4M2e-19S-Beihai32 and Beihai32-19S-4M2e (** *p* < 0.01). Notably, for 4M2e-19S-Beihai32 VLPs, no statistically significant differences compared to the PBS control group were detected, indicating that the immune response was effectively directed to the presented antigen rather than to the carrier protein.

### 3.5. The Recombinant Beihai32 Capsid Protein Carrying GFP at the N-Terminus Forms VLPs Presenting Functionally Active GFP on Their Surface

**Figure 6:**
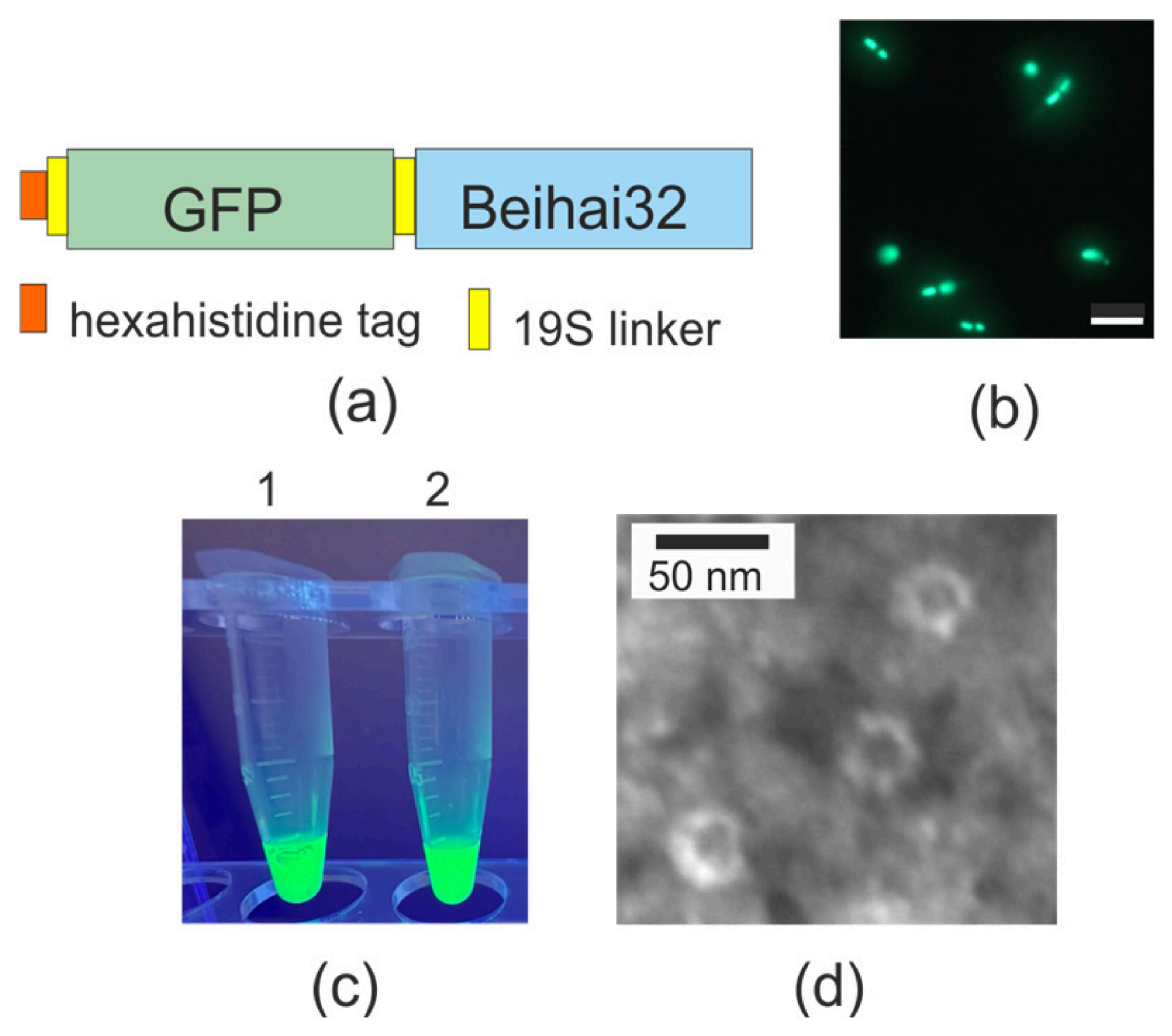
Expression and characterization of GFP-19S-Beihai32 protein. (**a**) Structure of the recombinant protein. (**b**) Fluorescent microscopy analysis of the induced culture. Scale bar 5 μm. (**c**) Visualization of fluorescence of solutions with equimolar concentration of GFP (1) and GFP-19S-Beihai32 (2) proteins. (**d**) Analysis of VLPs formed by recombinant GFP-19S-Beihai32 protein using TEM.

To evaluate the feasibility of inserting large proteins into the N-terminus of the Beihai32 CP protein, GFP (238 a.a.) was chosen as a model. GFP was genetically fused to the N-terminus of the Beihai32 CP protein via a 19S linker. The resulting gene was cloned into the pQE30 vector, allowing the expression of the recombinant protein with an N-terminal His-tag (Figure 6a). The recombinant protein was expressed at high levels in the *E. coli* and was found in both soluble and insoluble fractions. GFP was correctly folded in *E. coli* cells as revealed by fluorescent microscopy analysis of the induced culture (Figure 6b). Purification was performed from the soluble fraction of the cell lysate using metal affinity chromatography. After purification and dialysis, the GFP-19S-Beihai32 protein also exhibited characteristic fluorescence when irradiated with UV light (Figure 6c).

The assembly of the GFP-19S-Beihai32 protein into chimeric VLPs was analyzed using DLS and TEM. According to DLS measurements, the sample contained particles with an average size of approximately 40 nm, while TEM revealed VLPs with a diameter of approximately 35 nm (Figure 6d).

Structural modeling of the GFP-19S-Beihai32 recombinant protein using AlphaFold v.2.3.1 [24] predicted that the GFP moiety adopts a spatially distinct position relative to the CP (Supplementary Figure S2), supporting its potential display on the surface of assembled VLPs. Accordingly, GFP-19S-Beihai32 particles were expected to be accessible for recognition by GFP-specific antibodies.

**Figure 7:**
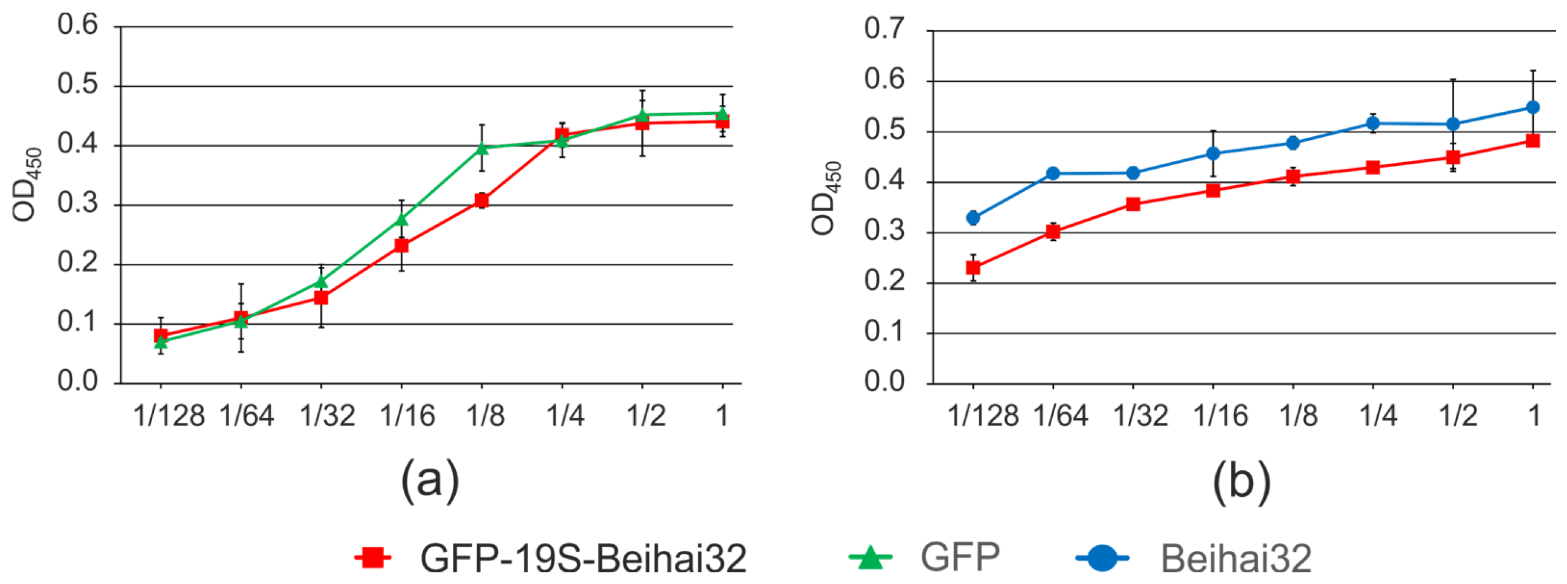
Antigenic properties of recombinant proteins GFP-19S-Beihai32 and GFP. Two-fold dilutions of GFP-19S-Beihai32 (starting with 15 μg/mL) and GFP (starting with 10 μg/mL) were applied to the plates, which were then incubated with anti-GFP antibodies (**a**). Two-fold dilutions of recombinant proteins GFP-19S-Beihai32 (starting with 30 μg/mL) and Beihai32 CP (starting with 10 μg/mL) were applied to the plates, which were then incubated with antibodies against Beihai32 CP (**b**). Protein concentrations were calculated based on the equimolar content of GFP or CP Beihai32 in the fusion protein molecule.

This prediction was confirmed by ELISA using anti-GFP antibodies, which demonstrated comparable binding efficiency to GFP-19S-Beihai32 VLPs and native GFP (Figure 7a). These findings indicate that GFP is exposed on the surface of the particles and retains its antigenic epitopes in the VLP context. At the same time, complete shielding of the CP core was not observed, as GFP-19S-Beihai32 VLPs remained capable of binding antibodies against Beihai32 CP, although less efficiently than unmodified Beihai32 VLPs (Figure 7b).

## 4. Discussion

The capsid protein of bacteriophage Beihai32 exhibits the ability to self-assemble into VLPs, making it a promising platform for the display of heterologous peptides. Previous studies have demonstrated that the C-terminal region of the CP can accommodate insertions of varying length without substantially impairing the self-assembly of VLP [16,17,18]. However, information on the possibility of heterologous insertions into the N-terminal region remains limited.

In the present study, we systematically investigated the tolerance of the N-terminal region of the Beihai32 CP to insertions of different lengths. Tandem repeats of the influenza A virus M2e peptide, ranging from one to four copies (23–119 a.a.), were used as model inserts. In addition, GFP (238 a.a.) was employed to assess the capacity of the capsid protein to accommodate larger proteins. This experimental design enabled a comprehensive evaluation of the impact of insert length and structural complexity on VLP assembly.

Hybrid capsid proteins were successfully expressed in *E. coli* cells, with one to four copies of the influenza A M2e peptide fused to the N-terminus either directly or via a flexible glycine-serine linker 19S. Surprisingly, proteins containing one copy of M2e (1M2e-Beihai32 and 1M2e-19S-Beihai32) were predominantly localized in inclusion bodies regardless of the presence of a linker, while fusion proteins containing two, three, or four M2e copies exhibited partial or predominant solubility. This phenomenon is likely related to the characteristics of the particular carrier protein, as it has not been described for other fusion partners. For example, the inclusion of one or several (2, 3, 4) copies of the M2e peptide in the hepatitis B virus core antigen did not impair the solubility of the fusion proteins or their ability to form VLPs [22,23].

In the engineering of VLPs for the display of heterologous peptides, flexible glycine-serine linkers are widely employed to spatially separate the carrier protein from the fused peptide. The use of such linkers reduces steric limitations and promotes proper protein folding, thereby increasing the likelihood of successful VLP assembly. For instance, several studies have demonstrated that, in the case of genetic fusion of antigens to the CP of bacteriophage AP205, the presence of a flexible linker preserves the ability of the protein to assemble into VLPs while ensuring the accessibility of the displayed epitope for antibody binding [11]. Similarly, it was demonstrated that the introduction of a flexible 12 a.a. long linker between the phage T7 capsid protein of the insert improves the functional properties of the fusion constructs [25]. However, the incorporation of flexible linkers does not universally result in increased protein solubility or improved VLP assembly. For example, in MS2-based VLP systems, the addition of a flexible linker did not enhance expression levels or assembly efficiency of particles carrying the dsRBD1 domain. These findings suggest that, within the AB loop region of the MS2 capsid protein, an insertion length of approximately 91 amino acids may represent an upper tolerance limit for heterologous domains [12]. In our M2e-Beihai32 CP fusion proteins, the inclusion of the 19S linker resulted in some improvement in the solubility of the recombinant proteins, as evidenced by their distribution between the soluble and insoluble fractions of the cell lysate. This effect was, however, relatively modest, probably because the Beihai32 CP termini are naturally localized on the particle surface.

TEM analysis of 4M2e-Beihai32 and 4M2e-19S-Beihai32 confirmed the formation of spherical VLPs with a diameter of approximately 30–35 nm, morphologically similar to native Beihai32 particles. Although the fusion proteins were approximately twice as long as the empty CP, the observed particle sizes were only slightly larger (Table 1). Apparently, the Beihai32 CP forms the microscopically visible dense core of the particle, while the inserts form the diffuse covering. This assumption is also supported by the observation that 4M2e-Beihai32 particles have a larger observed diameter (35.7 nm) than 4M2e-19S-Beihai32 particles (30.1 nm), in which the insert is expected to be more flexible due to the presence of the 19S linker. More pronounced differences between the sizes of 4M2e-Beihai32 (44.4 nm) and Beihai32 (27.7 nm) particles were observed using dynamic light scattering, which measures the hydrodynamic diameter rather than only the electron-dense core and in our case probably more accurately determines the actual particle size.

ELISA using anti-M2e antibodies demonstrated that the M2e peptide is accessible for binding in both N- and C-terminal fusions (4M2e-19S-Beihai32 and Beihai32-19S-4M2e), indicating its surface localization and preservation of native epitopes. This finding is of particular importance for vaccine design, as the immunogenicity of VLP-based platforms critically depends on the spatial presentation and conformational integrity of the displayed antigen. Surface exposure of the M2e peptide in VLPs suggests its correct orientation and the absence of substantial steric hindrance, thereby facilitating efficient recognition by the immune system.

At the same time, complete masking of the carrier protein within VLPs from anti-CP antibodies was not observed. This may be attributed to the structural organization of the capsid protein, in which both the N- and C-termini extends outward from the particle surface [14], while the inserted peptides were linked via a long flexible 19S linker, leaving the central region of the particle accessible to antibodies directed against the CP. Perhaps shortening the linker will allow for a more compact VLP structure in which the attached epitopes will better shield the CP core of the particle.

Immunization of mice with 4M2e-19S-Beihai32 and Beihai32-19S-4M2e particles induced high titers of M2e-specific IgG antibodies regardless of the insertion site. At the same time, differences were observed in the induction of antibodies against the carrier protein. Particles bearing N-terminal M2e insertions did not elicit a significant anti-capsid antibody titers compared to the control group, whereas C-terminal fusions induced a pronounced immune response against the CP. These findings suggest that N-terminal insertions may partially shield immunodominant regions of the CP, thereby reducing its immunogenicity. From a practical perspective, this represents an important advantage, as the reduction in anti-carrier response may facilitate a more targeted immune response toward the target antigen and allow multiple immunizations to be carried out without induction of anti-carrier reactions.

Such problems have previously been reported for VLPs based on the CP of bacteriophage MS2 [26]. Despite the high efficiency of this platform for peptide antigen display, a significant drawback was the induction of a strong immune response directed against the MS2 capsid protein itself rather than the inserted epitopes. This can result in rapid recognition and clearance of the particles upon repeated immunizations using the same carrier. It has also been shown that increasing the size of inserted sequences is associated with a reduction in the fraction of chimeric VLPs recognized by immobilized polyclonal IgG against the MS2 capsid protein [26]. This effect indicates that larger insertions can partially or completely mask carrier protein epitopes, thereby reducing their accessibility to antibodies.

Finally, to evaluate the ability of the N-terminus of the Beihai32 CP to accommodate large peptide insertions, a recombinant fusion protein bearing GFP fused to the N-terminus (GFP-19S-Beihai32) was obtained. TEM analysis of GFP-19S-Beihai32 confirmed the formation of spherical VLPs with a diameter of approximately 35 nm, morphologically similar to native Beihai32 particles. Like in the case of M2e fusion, the average diameter of the chimeric GFP-19S-Beihai32 VLPs was comparable to that of native Beihai32 particles, despite an almost twofold increase in molecular weight due to the insertion of GFP. The Beihai32 CP probably formed a compact, electron-dense core of the particle, whereas the GFP domain was located peripherally and forms a less dense outer layer. A similar pattern was observed in our previous study involving C-terminal presentation of GFP by the Beihai32 CP [18]. Additional evidence supporting correct folding of the GFP domain in the native conformation was provided by the presence of characteristic fluorescence in the green region of the spectrum when irradiated with ultraviolet light. Analysis of the antigenic properties of GFP-19S-Beihai32 VLPs by ELISA demonstrated efficient binding to GFP-specific antibodies. These results indicated that the fluorescent domain is displayed on the surface of the assembled VLPs and remains accessible to antibodies.

The N-terminal region of the Beihai32 capsid protein was found to tolerate long insertions without losing the ability to self-assemble into VLPs. This contrasts with several other bacteriophage-based platforms, where the introduction of long insertions disrupts particle assembly. For example, in bacteriophage AP205, N-terminal insertions longer than 39 a.a. disrupted proper assembly, whereas C-terminal modifications were generally more tolerant [11]. In the case of bacteriophage MS2, in addition to insertions into the AB loop region, modifications of both the N- and C-terminal regions are possible. However, both the N- and C-termini exhibit limited tolerance to insertions. It was shown that peptides of only 10–27 a.a. can be fused to the N-terminus of the CP without disrupting particle assembly, while even short insertions at the C-terminus severely disrupt capsid assembly and are only possible using methods to dramatically reduce display valency [27]. Up to 91 a.a. peptides can be inserted into the AB-loop region of MS2 CP [12].

Our results demonstrate that the Beihai32 CP represents a versatile platform for the construction of VLPs. Both the N and C termini of the CP can be used to insert large peptides without disrupting the self-assembly of the fusion proteins into the VLPs, with the resulting chimeric VLPs presenting the inserted peptides on their surface. The ability to incorporate insertions at both ends of the CP molecule expands the possibilities for the design of chimeric VLPs, presenting multiple peptide antigens simultaneously. Further studies will be focused on the possibility of simultaneously fusing target peptides to both the N and C termini of a single Beihai32 CP molecule, which should increase the density of antigen presentation on the surface of VLP. Overall, our findings expand the current understanding of the structural plasticity of ssRNA bacteriophage-based VLPs and highlight their potential for applications in vaccine development and biotechnology.

## 5. Conclusions

The CP of ssRNA phage Beihai32 could be used as a carrier for the presentation of relatively large antigens on the surface of recombinant VLPs via genetic fusion of the antigen to either N- or C-terminus of CP. Hybrid CPs with one to four copies of the influenza A virus M2e peptide fused to the N-terminus were expressed in *Escherichia coli*. Fusion proteins containing four copies of M2e self-assembled into spherical VLPs, displaying the inserted peptides on the surface. Immunization of mice with chimeric VLPs induced high titers of M2e-specific antibodies. Notably, in contrast to the C-terminal fusions, the N-terminal insertion masked the carrier protein and prevented the induction of anti-carrier antibody response. The N-terminus of Beihai32 CP is tolerant of large insertions, as demonstrated by the successful incorporation of GFP (238 a.a.) which was displayed on the particle surface and retained its fluorescent activity. Overall, the Beihai32 CP is a versatile platform for the presentation of peptide epitopes and full-length protein antigens, supporting its potential application in VLP-based vaccine design.

## Figures and Tables

**Table 1:** The sizes of VLPs based on the capsid protein of phage Beihai32.

Method	4M2e-Beihai32	4M2e-19S-Beihai32	Beihai32-19S-4M2e	Beihai32
DLS ^1^	44.4 ± 4.1 nm	32.8 ± 2.4 nm	29.8 ± 4.1 nm	27.7 ± 3.0 nm
TEM ^1^	35.7 ± 3.4 nm	30.1 ± 3.6 nm	33.3 ± 2.7 nm	27.3 ± 3.3 nm

^1^ Average and standard deviation of ten measurements.

## Data Availability

The original contributions presented in this study are included in the article. Further inquiries can be directed to the corresponding author.

## Supplementary Materials

The following supporting information can be downloaded at https://www.mdpi.com/article/10.3390/nano16150932/s1. Figure S1: Purification of recombinant proteins. Purified proteins were analyzed by SDS-PAGE. **M** is the molecular weight marker (kDa); *1*, 4M2e-Beihai32; *2*, 4M2e-19S-Beihai32; *3*, Beihai32-19S-4M2e. Figure S2: Predicted structures of recombinant proteins. The structures of monomeric proteins were predicted using AlphaFold v.2.3.1 and visualized using the SWISS-MODEL server. Note that similarly to GFP-19S-Beihai32, the hexahistidine tag and the 19S linker were fused to the N-terminus of GFP.

## Abbreviations

The following abbreviations are used in this manuscript:

VLP	virus-like particle
M2e	extracellular domain of the transmembrane protein M2
CP	capsid protein
ssRNA	single-stranded RNA

## References

[1] Nooraei S., Bahrulolum H., Hoseini Z.S., Katalani C., Hajizade A., Easton A.J., Ahmadian G. (2021). Virus-like Particles: Preparation, Immunogenicity and Their Roles as Nanovaccines and Drug Nanocarriers. J. Nanobiotechnol..

[2] Kheirvari M., Liu H., Tumban E. (2023). Virus-like Particle Vaccines and Platforms for Vaccine Development. Viruses.

[3] Tars K., Witzany G. (2020). ssRNA Phages: Life Cycle, Structure and Applications. Biocommunication of Phages.

[4] Dai X., Li Z., Lai M., Shu S., Du Y., Zhou Z.H., Sun R. (2017). In Situ Structures of the Genome and Genome-Delivery Apparatus in a Single-Stranded RNA Virus. Nature.

[5] Dong Y., Zhang G., Huang X., Chen L., Chen H. (2015). Promising MS2 Mediated Virus-like Particle Vaccine against Foot-and-Mouth Disease. Antivir. Res..

[6] Zhai L., Peabody J., Pang Y.-Y.S., Schiller J., Chackerian B., Tumban E. (2017). A Novel Candidate HPV Vaccine: MS2 Phage VLP Displaying a Tandem HPV L2 Peptide Offers Similar Protection in Mice to Gardasil-9. Antivir. Res..

[7] Zhai L., Yadav R., Kunda N.K., Anderson D., Bruckner E., Miller E.K., Basu R., Muttil P., Tumban E. (2019). Oral Immunization with Bacteriophage MS2-L2 VLPs Protects against Oral and Genital Infection with Multiple HPV Types Associated with Head & Neck Cancers and Cervical Cancer. Antivir. Res..

[8] Tumban E., Peabody J., Peabody D.S., Chackerian B. (2011). A Pan-HPV Vaccine Based on Bacteriophage PP7 VLPs Displaying Broadly Cross-Neutralizing Epitopes from the HPV Minor Capsid Protein, L2. PLoS ONE.

[9] Cohen A.A., Gnanapragasam P.N.P., Lee Y.E., Hoffman P.R., Ou S., Kakutani L.M., Keeffe J.R., Wu H.-J., Howarth M., West A.P. (2021). Mosaic Nanoparticles Elicit Cross-Reactive Immune Responses to Zoonotic Coronaviruses in Mice. Science.

[10] Kirsteina A., Akopjana I., Bogans J., Lieknina I., Jansons J., Skrastina D., Kazaka T., Tars K., Isakova-Sivak I., Mezhenskaya D. (2020). Construction and Immunogenicity of a Novel Multivalent Vaccine Prototype Based on Conserved Influenza Virus Antigens. Vaccines.

[11] Tissot A.C., Renhofa R., Schmitz N., Cielens I., Meijerink E., Ose V., Jennings G.T., Saudan P., Pumpens P., Bachmann M.F. (2010). Versatile Virus-Like Particle Carrier for Epitope Based Vaccines. PLoS ONE.

[12] Dang M., Wu L.J., Zhang S.R., Zhu J.R., Hu Y.Z., Yang C.X., Zhang X.Y. (2023). MS2 Virus-like Particles as a Versatile Peptide Presentation Platform: Insights into the Deterministic Abilities for Accommodating Heterologous Peptide Lengths. ACS Synth. Biol..

[13] Liekniņa I., Kalniņš G., Akopjana I., Bogans J., Šišovs M., Jansons J., Rūmnieks J., Tārs K. (2019). Production and Characterization of Novel ssRNA Bacteriophage Virus-like Particles from Metagenomic Sequencing Data. J. Nanobiotechnol..

[14] Rūmnieks J., Liekniņa I., Kalniņš G., Šišovs M., Akopjana I., Bogans J., Tārs K. (2020). Three-Dimensional Structure of 22 Uncultured ssRNA Bacteriophages: Flexibility of the Coat Protein Fold and Variations in Particle Shapes. Sci. Adv..

[15] Shishovs M., Rumnieks J., Diebolder C., Jaudzems K., Andreas L.B., Stanek J., Kazaks A., Kotelovica S., Akopjana I., Pintacuda G. (2016). Structure of AP205 Coat Protein Reveals Circular Permutation in ssRNA Bacteriophages. J. Mol. Biol..

[16] Vasyagin E.A., Zykova A.A., Mardanova E.S., Nikitin N.A., Shuklina M.A., Ozhereleva O.O., Stepanova L.A., Tsybalova L.M., Blokhina E.A., Ravin N.V. (2024). Influenza A Vaccine Candidates Based on Virus-like Particles Formed by Coat Proteins of Single-Stranded RNA Phages Beihai32 and PQ465. Vaccines.

[17] Vasyagin E.A., Zykova A.A., Blokhina E.A., Ozhereleva O.O., Stepanova L.A., Shuklina M.A., Klotchenko S.A., Mardanova E.S., Ravin N.V. (2025). Chimeric Virus-like Particles Formed by the Coat Proteins of Single-Stranded RNA Phages Beihai32 and PQ465, Simultaneously Displaying the M2e Peptide and the Stalk HA Peptide from Influenza a Virus, Elicit Humoral and T-Cell Immune Responses in Mice. Vaccines.

[18] Zykova A.A., Mardanova E.S., Blokhina E.A., Ravin N.V. (2026). Capsid Protein of Bacteriophage Beihai32 as a Platform for Construction of Virus-Like Particles Displaying Foreign Polypeptides. Mol. Biol..

[19] Liekniņa I., Černova D., Rūmnieks J., Tārs K. (2020). Novel ssRNA Phage VLP Platform for Displaying Foreign Epitopes by Genetic Fusion. Vaccine.

[20] Vasyagin E.A., Mardanova E.S., Ravin N.V. (2025). Virus-like Particles Formed by the Coat Protein of the Single-Stranded RNA Phage PQ465 as a Carrier for Antigen Presentation. Molecules.

[21] Robinson C.R., Sauer R.T. (1998). Optimizing the Stability of Single-Chain Proteins by Linker Length and Composition Mutagenesis. Proc. Natl. Acad. Sci. USA.

[22] De Filette M., Min Jou W., Birkett A., Lyons K., Schultz B., Tonkyro A., Resch S., Fiers W. (2005). Universal Influenza A Vaccine: Optimization of M2-Based Constructs. Virology.

[23] Ravin N.V., Blokhina E.A., Kuprianov V.V., Stepanova L.A., Shaldjan A.A., Kovaleva A.A., Tsybalova L.M., Skryabin K.G. (2015). Development of a Candidate Influenza Vaccine Based on Virus-like Particles Displaying Influenza M2e Peptide into the Immunodominant Loop Region of Hepatitis B Core Antigen: Insertion of Multiple Copies of M2e Increases Immunogenicity and Protective Efficiency. Vaccine.

[24] Abramson J., Adler J., Dunger J., Evans R., Green T., Pritzel A., Ronneberger O., Willmore L., Ballard A.J., Bambrick J. (2024). Accurate Structure Prediction of Biomolecular Interactions with AlphaFold 3. Nature.

[25] Hufziger K.A., Farquharson E.L., Werner B.G., Chen Q., Goddard J.M., Nugen S.R. (2022). In Vivo Capsid Engineering of Bacteriophages for Oriented Surface Conjugation. ACS Appl. Bio Mater..

[26] Brown W.L., Mastico R.A., Wu M., Heal K.G., Adams C.J., Murray J.B., Simpson J.C., Lord J.M., Taylor-Robinson A.W., Stockley P.G. (2002). RNA Bacteriophage Capsid-Mediated Drug Delivery and Epitope Presentation. Intervirology.

[27] Tumban E., Peabody J., Tyler M., Peabody D.S., Chackerian B. (2012). VLPs Displaying a Single L2 Epitope Induce Broadly Cross-Neutralizing Antibodies against Human Papillomavirus. PLoS ONE.

