# Supplementary material for: Virus-like Particles Derived from Bacteriophage Beihai32 as a Versatile Carrier for Displaying Large Peptide Antigens
Source: Nanomaterials (Basel). 2026 Jul 29;16(15):932. doi: 10.3390/nano16150932 (PMC13468365; doi:10.3390/nano16150932)
Supplement: Supplementary file 1 [file nanomaterials-16-00932-s001.zip › Supplementary Figure S1.pdf]

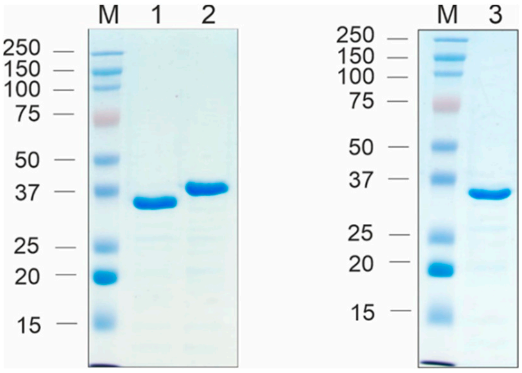

Supplementary Figure S1. Purification of recombinant proteins.
Purified proteins were analyzed by SDS-PAGE. **M** is the molecular weight marker (kDa); *1*, 4M2e-Beihai32; *2*, 4M2e-19S-Beihai32; *3*, Beihai32-19S-4M2e.
