# Supplementary material for: Virus-like Particles Derived from Bacteriophage Beihai32 as a Versatile Carrier for Displaying Large Peptide Antigens
Source: Nanomaterials (Basel). 2026 Jul 29;16(15):932. doi: 10.3390/nano16150932 (PMC13468365; doi:10.3390/nano16150932)
Supplement: Supplementary file 1 [file nanomaterials-16-00932-s001.zip › Supplementary Figure S2.pdf]

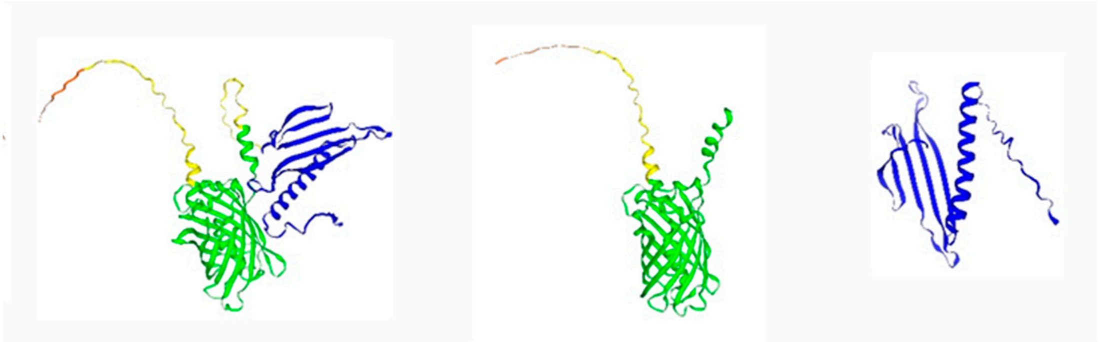

GFP-19S-Beihai32 GFP Beihai32

Supplementary Figure S2. Predicted structures of recombinant proteins.
The structures of monomeric proteins were predicted using AlphaFold v.2.3.1 and visualized using the SWISS-MODEL server. Note that similarly to GFP-19S-Beihai32, the hexahistidine tag and the 19S linker were fused to the N-terminus of GFP.
